# Degeneration and Decay of the Oral Tissues from Lack of Exercise

**Published:** 1899-03

**Authors:** S. B. Dewey

**Affiliations:** Cleveland, O.


ARTICLE IX.

### DEGENERATION AND DECAY OF THE ORAL TISSUES FROM LACK OF EXERCISE.

S. B. DEWEY, D. D. S., M. D., CLEVELAND, O.

That the oral tissues are degenerating must be apparent to the most casual observer. The lack of full development of the jaws and a symmetry in the arrangement of the teeth is due to degeneration or want of normal force to reproduce the kind to its full standard. The first stages of development may be retarded owing to the feeble state of the nervous system, which controls assimilation and nutrition. The impress upon the embryo will be more or less apparent throughout the developmental period, but if such means as are at our command be used to overcome this condition, much improvement will be noted.

The jaw develops from before backwards; therefore, jaws not exercised by mastication would be less in length than one which had been well exercised.

One of the chief factors in the arrest of dovelopment of the jaws is the want of auxiliary exercise. Blood is not carried to the parts to nourish the bones. The teeth suffer in a like manner. The lack of lateral motion from little work given the teeth, the alveolar process will be insufficiently developed.

Jaws and teeth that are not used to grind the food will be imperfectly developed, weak processes, tooth socket poorly nourished, peridental membrane and gums loose in structure, readily yielding to destructive influences, lack of development because we fail to use it for its normal function, therefore fails to develop for want of use or requirement.

If man's food habits are such that he requires but little of his dental organs, they will develop accordingly.

As soon as the being passes from the stage of infancy into childhood (infancy ends with completion of first dentition), the mouth is equipped with a mill that was designed to grind coarse food into a fine pasty mass, that may be appropriated by the economy for the upbuilding and maintenance of the body, and if, on entering childhood, the child were taught that all foods should be thoroughly masticated, and incorporated with saliva before passing into the stomach, the advantages gained are threefold. First, the chewing would give exercise to the jaws and teeth, promoting their growth and perfecting the type; secondly, the perfect preparation of the food for the future digestive process would be sure to insure its assimilation; thirdly, under these conditions the nutritive changes would be in balance, and structures would be builded that would better withstand unfavorable environment.

The tendency of both man and child is to discard all foods that are hard and not ready to be hastily swallowed. The diet should consist of such food as requires chewing and will furnish the proper nourishment to the developing teeth and surroundings, and in later life must serve to clean them and keep the tooth socket supplied with new blood, that its repair may at least equal the waste. In the unused organs the waste is greater than the repair, and as a result we have breaking down of the attachments, pus formations and their loss.

Nature designed that the teeth should be cleaned by chewing solid food, and Dr. Black says: "Keep the margins of the fillings in the interproximate space clean by frequent excursions of food over them." Pray tell us how we are going to do this unless our diet is composed of food that requires chewing, that these excursions may be made.

As dentists we are appalled at the number of children of tender years who require a large number of fillings in their permanent teeth, who on examination present teeth and gum margins thickly coated with the soft remains of starchy food in an active state of fermentation. Would this be so if the child lived on food that required chewing?

The thorough mastication of the food means to the child clean teeth, free from decay, perfectly developed jaws, with a minimum of irregularity, and the building up of a tooth socket and gum tissue of such quality that phagedenic pericementitis will be a thing unknown in middle and later life. Tough beef and the hard-baked loaf would prove enemies of dyspepsia, malnutrition, dental decay and irregularity, and the friend of mankind and his progeny. How often are we disgusted with our older patients who have applied for relief from so-called pyorrhea alveolaris, and after we have spent much time in scraping and cleaning to free the teeth and gums of the objectionable accumulations, to have them return after an interval of two or three days with the mouth again reeking with the debris of the past few meals

Our patients rely too much on our skill and ability to relieve them of their difficulties, instead of by careful effort trying to avoid those irregularities they know lead to diseased conditions. Whoever knew of a real case of phagedenic pericementitis among those who use the masticatory organs fully ? I have yet to see the first case, and my experience is not a little in this line. Did you ever see the diseases in a tobacco chewer's mouth ? You may find pockets with accumulations crowded therein, but no pus; at least this is my experience.

Some writer on irregularities of the teeth says that irregularity does not exist in the tobacco-chewer's mouth. The habit corrects or prevents it. For some time past I have taken pains to inquire into the habits of those demanding treatment for so-called pyorrhea, and find it the rule, with no exceptions, that the victim bolts his food, which consists largely of mush and sops. Just recently, on asking a lady who is under treatment two or three times a year, as to her food habits, she replied: "My sister-in-law says she never saw a family so given to sopping food as ours." The conditions present in her mouth verified the truth of her statement.

In the many recent discussions of this vexed topic much has been said about the teeth being the farthest from the centers of nutrition, and therefore suffer in consequence. This being so, the more the need of pressing the teeth into active service, requiring a certain amount of work at each meal, that new blood may be brought to the parts, thus aiding in the supply of nutritive material. Much stress is also placed on the importance of perfect assimilation of the ingested food that we may have perfect nutrition.

Did I hear a voice advocating the more thorough mastication of the food, that it may be easily digested and readily assimilated ?

We should be teachers as well as healers, ever ready to detect abnormal conditions and vicious habits and offer the proper advice for their correction.

We will find it labor lost trying to reform adult man, but we can place those matters in such light that those having the care of children will feel their importance and enforce their application. In an Eastern kindergarten children are required to bring a luncheon of bread and butter and fruit, which is eaten in the presence of the teachers, and they are instructed to see that each child eats the crusts of bread. It would seem, in this case at least, that the teachers, instead of the dentists, had taken the initiative in this important matter. The mouths of fever patients do not become free from their coatings until they begin to eat solid food.

The coatings on the tongue and mucous membrane contain bacteria which will excite fermentation in food particles lodging on the teeth. The chewing of coarse food will tear off this film of bacteria that may coat the teeth. Teeth used daily in mastication do not have the accumulation of calculus that are to be seen on the unused organs. Examples are found in mouths where one side is disabled. Dr. Talbot says : "If the mouth and teeth were kept absolutely clean, so that chemical changes could not take place, the teeth would certainly never decay, no matter how imperfectly the enamel might be developed. If the gums be stimulated in a proper manner and kept in a healthy condition, they are decidedly less liable to become infected with pyorrhea." He did not suggest a means of obtaining this desired end. Let me say that proper mastication will keep the mouth clean, nutrition of the teeth and gums more perfect and that the act will furnish the proper stimulus to keep the gums and tooth socket in a healthy condition. The beauty and health of the teeth depend on the perfect harmony of nutrition and absorption, waste and repair.

It is better to rely on the chewing to keep the teeth and mucous membrane clean, than upon the use of drugs. Nature never designed the mouth to be the testing ground for all the antiseptics that the chemist might produce.

The constant use of antiseptics lowers the vitality of tissues and renders them readily assailable bv destructive force.

Atrophy of the jaw from non-use or faulty nutrition is one of the neurctic manifestations. Over development of the brain and nervous system a tendency in modern life. The most rapid growth of the brain occurs during the first seven years, and if we over-stimulate this growth it is at the expense of the osseous system. The most vascular parts are first served with nutritive elements, and if there is not enough to go around the parts farthest from the seat of nutrition (the bones and teeth), the least vascular, must consequently suffer from this extra brain development. Well digested and assimilated food must produce a well nourished nervous system, a normal nervous system, a well developed body in all its parts.

The time that imperfect development of the maxillary bones begin to show and irregularity of the teeth is apparent, is between the ages of six and twelve years. At a time when our children are living on a diet consisting largely of cooked ground grains, or mush, which require little or no chewing, a habit of diet which robs the jaws and teeth of the exercise which the chewing of more solid food would give them. If habits of exercising the jaws and teeth were acquired at this period, better development would be obtained, and much irregularity would be averted.

"It is said the wholesale extraction of the first molar in the past has caused arrest of development of the alveolar process as well as of the maxillary bones, for the process and jaws depend for their development largely on the function of the teeth, their articulation and their motion stimulating nutrition and enlarging the arch. The horizontal portion of the lower jaw will be improperly developed because function, one of the most important means of development, is lost, and insufficient room is left for the second and third molars."

If we must needs keep this tooth for work, that the jaw may be properly developed, then let us give all the teeth all the exercise nature designed them to have, and surely we will have jaws and teeth in greater harmony.

As for food that is prepared at the factory and ready to be swallowed at once, why, we will bequeath that to the unfortunate who wear factory teeth that will not decay, and have not the peridental membrane to degenerate, therefore cannot suffer from lack of exercise. And as a last argument for the more thorough mastication of the food, let me remind you that it is now claimed that the human being can live without a stomach, and should man through misfortune or degeneracy be deprived of this supposed important organ, then surely "the mill must grind exceedingly fine" and the provender be well mixed with saliva before it starts on its shortened route.—*Indiana Dental Journal.*

